# Deciphering the Physical Characteristics of Ophthalmic Filters Used in Optometric Vision Therapy

**DOI:** 10.3390/healthcare12212177

**Published:** 2024-10-31

**Authors:** Danjela Ibrahimi, Marcos Aviles, Guillermo Valencia Luna, Juvenal Rodriguez Resendiz

**Affiliations:** 1 Facultad de Medicina, Universidad Autónoma de Querétaro, Santiago de Querétaro 76010, Mexico; danjela.ibrahimi@uaq.mx; 2 Brain Vision & Learning Center, Misión de Capistrano 117, Juriquilla, Santiago de Querétaro 76226, Mexico; 3 Facultad de Ingeniería, Universidad Autónoma de Querétaro, Santiago de Querétaro 76010, Mexico; 4 Laboratorio de Propiedades Ópticas de los Materiales de la Dirección Óptica y Radiometría, del Centro Nacional de Metrología, El Marques 76246, Mexico; gvalenci@cenam.mx

**Keywords:** ocular treatment, visual health, monochromatic filters, visual therapy

## Abstract

Background: This paper aimed to measure and characterize eleven monochromatic filters and twenty-two combinations used empirically to treat patients with visual dysfunctions to propose enhanced protocols based on solid evidence. Their wavelength, transmittance, and relative sensitivity were defined on the retinal cone cells. Methods: A double-beam UV-VIS-NIR spectrophotometer, VARIAN brand, Cary 5000 model, owned by the National Center of Metrology, with high precision and accuracy, was used to characterize all filters. Filters were purchased from Optomatters Corporation, Belgium. Results: When two or three filters are combined, their transmittance and relative sensitivity on the retinal cone cells decrease regardless of wavelength. As a result, the efficiency of combined filters may decrease during treatments. Additionally, most filters and combinations, regardless of the wavelength, transmit a considerable percentage of light from the red spectrum. A depressant is the best monochromatic filter, and Upsilon–Neurasthenic is the strongest combination to stimulate blue cone cells. In contrast, Stimulant and Delta–Theta are best for red and green cone cells. Mu–Delta and Mu–Theta can be interchangeable, as well as Alpha–Delta and Alpha–Theta. Conclusions: Results suggest that the current phototherapy treatment protocol must be deeply revised, and the number of filters and combinations should be reduced to reduce costs and time and boost efficiency.

## 1. Introduction

This paper aimed to characterize eleven monochromatic spectral filters and twenty-two combinations empirically used to treat patients with visual dysfunctions. For the first time, properties such as wavelengths, transmissions, and relative sensitivities are measured and presented to propose innovative, scientifically based treatments.

Currently, light stimulation and phototherapy utilizing a combination of two or more spectral filters have been empirically applied as part of the therapeutic process for patients with visual dysfunctions [1,2]. In clinical settings, the choice of filters is guided by the patient’s medical history, symptoms, and clinical findings, following the protocol established by the College of Syntonic Optometry (CSO) for patients with visual deficiencies [1,3]. These monochromatic filters transmit light from the blue to the red spectrum, representing the visible light spectrum (VLS). Their effect on brain activity begins in cells that respond to luminance, color, or both, located in the primary visual cortex (V1) [4], and extends to additional cortical regions through the extensive neural network and brain connectome.

Moreover, the theory posits that low-energy, long-wavelength light (red) stimulates the sympathetic nervous system; mid-length wavelengths (green) balance physiology; and high-energy, short-wavelength light (blue) activates the parasympathetic nervous system, modulating the autonomic nervous system through the retinohypothalamic tract (i.e., the non-visual pathway of light perception) and its projections to subcortical and cortical regions [5]. Therefore, the neural activity of patients with neurodevelopmental and neurological conditions may be altered and enhanced by wavelength-dependent processes and other energy-based stimulation [6].

Visual-evoked potentials (VEPs) have demonstrated that monochromatic filters can alter VEP responses by influencing axonal activation and the number of fibers that become connected [7,8]. Similarly, quantitative electroencephalography (qEEG) studies have shown that light stimulation and phototherapy, utilizing a combination of two or more spectral filters, can modulate Alpha waves, interhemispheric connections, the anteroposterior gradient, and brain coherence [9,10]. Additionally, functional connectivity patterns in brain networks of neurotypically developed individuals, as measured by fMRI, have been found to be light-dependent [11].

The earliest clinical studies using monochromatic and combined filters reported changes in form visual fields in reading disabled children and in certain visual and cognitive functions [12,13].

A more recent study which combined clinical and neuroimaging data showed the positive impact of spectral filters in patients with strabismus and amblyopia on the angle of deviation, visual acuity, stereopsis degrees, and visual fields [9]. Moreover, the modulation effect of the spectral filters on the brain connectome can be extended to patients with traumatic brain injuries [14], children diagnosed with visual stress [15], patients with symptoms of visual processing disorder and migraine [16,17], etc. Hence, their impact on brain activity is undeniable. However, there is an essential difference among these papers; the filters’ wavelengths and transmittances differ substantially. In the field of neuro-optometry, light stimulation and phototherapy have become a substantial part of the visual treatment of patients with visual dysfunctions, and filters are used based on a specified protocol [1,3], but their properties are not available to clinicians.

As a consequence, there needs to be solid scientific evidence to support their use. However, light stimulation using these spectral filters became popular in the late 1980s and early 1990s, but it changed the understanding of the mechanism by which light stimulation affects patients with visual disorders, and the cortical electrical response to such stimulation was a paper published in 1996 [18].

Since then, the protocol of filters used has been updated several times based on empirical and clinical evidence by the CSO, but information is mostly transmitted during seminars and conferences or case reports published in the CSO journal.

In our previous study, wavelength and transmittance of six monochromatic filters were measured [8], and their effect was analyzed through VEPs, which raised more questions concerning the vast number of combinations used in visual therapy. Additionally, the use of these specific twenty-two combined filters, their impact on the retinal cone cells of the retina, as well as their different impact on the cerebral cortex despite patients presenting the same visual dysfunction [9,10] remain unclear. Consequently, we need more scientific data to personalize treatments and guarantee results. Another point worth mentioning is the elevated purchasing cost of these filters and the uncertainty of their effectiveness. As for every scientific unknown, identifying the problem and analyzing its component leads to new knowledge, which is the goal of this paper. Our scientific contribution is to understand their physical characteristics in order to properly apply them and propose improved protocols in the forthcoming future. This would not only reduce costs, time and boost the effect of the treatment, but also provide a solid scientific base.

The structure of this work is as follows: Section 2 details the methods applied and presents the results and findings obtained. Finally, Section 3 outlines the areas covered within the scope of this work.

## 2. Methods and Results

This is a descriptive quantitative study that measures the wavelength, transmittance, and relative sensitivity on the retinal cone cells of eleven monochromatic filters and twenty-two combinations used empirically in the clinical practice of visual health professionals to treat patients with visual dysfunctions. This paper aims to define their physical properties to propose enhanced protocols based on solid scientific evidence. Six high-energy, short-wavelength filters (Omega, Lambda, Pi, Neurasthenic, Upsilon, Depressant) and five low-energy, long-wavelength filters (Alpha, Delta, Theta, stimulant) used in clinical practice are presented. Mu, on the other hand, is a mid-wavelength filter. Combinations result from combining two or three monochromatic filters. Filters were purchased from Optomatters Corporation, Belgium.

Results section is divided into 3 sub-sections:

- Section 2.1 presents the wavelength and transmittance of all monochromatic and combined filters.
- Section 2.2 measures the response of the three cone cells of the retina to each filter, represented by the relative sensitivity.
- Section 2.3 compares five glass and polymer filters.

### 2.1. Characterization of the Filters

The spectral transmittance of each monochromatic filter was measured using a double-beam UV-VIS-NIR spectrophotometer, model Cary 5000, manufactured by VARIAN and owned by the National Center of Metrology. This device provides high precision and accuracy and is calibrated for transmittance values (expressed as percentages) and wavelengths (expressed in nanometers). These measurements allow for determining the transmittance values as a percentage for each wavelength, ranging from 380.0 nm to 750.0 nm, with increments of 0.5 nm. Both sets of measurements are traceable to the National Spectral Transmittance, Absorbance, and Reflectance Standard maintained by the NCM [19]. Additional details on the calibration uncertainty for transmittance and wavelength measurements, as well as the characterization of the filters, can be found in the methodology section of our previous study [8]. Table 1 presents the range of the Visible Light Spectrum (VLS), while Table 2 illustrates the wavelength range of the monochromatic filters used in visual therapy.

**Figure 1:**
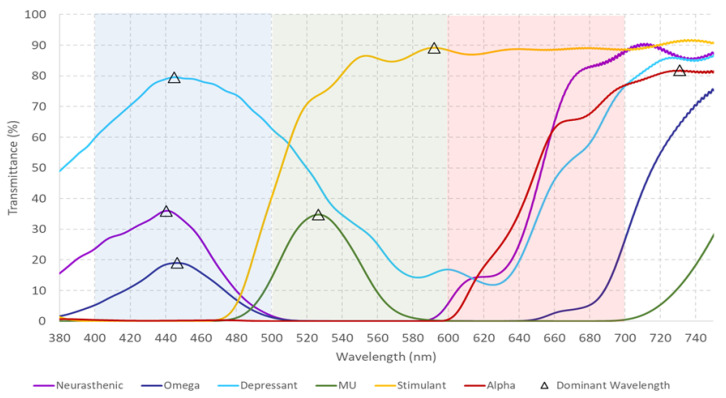
Spectral transmittance measurements (ranging from 380 to 750 nm) for six monochromatic filters, with the dominant wavelength marked by a triangle. The colors displayed in the graphs correspond to the actual colors of the filters. The dominant wavelength, defined as “the wavelength with the highest transmittance in the measured spectra”, is within the ranges specified in the Visible Light Spectrum (Table 1) and aligns with the perceived color of each filter. Reprinted from [8].

**Figure 2:**
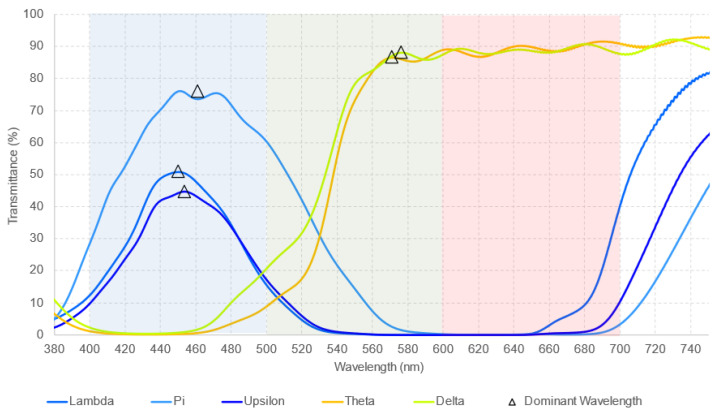
Measured spectral transmittance (from 380 to 750 nm) for the dominant wavelength of five additional monochromatic filters, with each dominant wavelength represented by a triangle. The colors used in the graphs are selected to best represent the actual colors of the filters. The dominant wavelength, defined as “the wavelength with the highest transmittance in the measured spectra”, falls within the specified ranges of the Visible Light Spectrum (Table 1) and corresponds to the perceived color of each filter.

Figure 1 presents the transmittance obtained with the six monochromatic filters previously reported in [8]. In contrast, Figure 2 shows the transmittance corresponding to the analysis of the five new monochromatic filters.

Afterward, to generate greater knowledge and understanding of these filters, the VLS was analyzed in its seven basic wavelength ranges, ranging from violet (360 to 440 nm) to red (620 to 780 nm), and the average transmittance of each monochromatic filter and their combinations in every wavelength range was measured. Table 3 shows the average transmittance of monochromatic filters, while Table 4, Table 5, Table 6 and Table 7 refer to all combinations used to treat patients with visual dysfunctions.

Table 3 indicates that the Depressant and Pi filter dominates the rest of the monochromatic filters, which transmit light from the blue spectrum. Likewise, Stimulant, Delta, and Theta filters, which transmit light from the red spectrum similarly, show a higher transmittance than Alpha.

**Figure 3:**
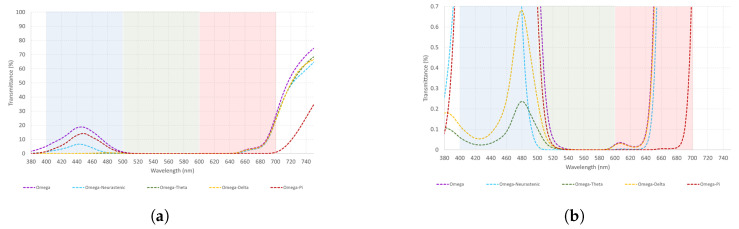
(**a**,**b**) The measured spectral transmittance (from 380 to 750 nm) of Omega combinations in two different scales for its better understanding. The colors on the graphs are chosen to represent in the best possible way the colors perceived through each combination.

Omega is a short-wavelength filter. Table 4 shows how Omega combinations show a higher light transmittance to the red spectrum than blue, with Omega–Pi being the best combination to transmit light from the blue spectrum. This means that Omega loses power when combined with other monochromatic filters. A graphical representation of these data is illustrated in Figure 3a,b.

Taking into consideration that Omega and its combinations are used to transmit light from the blue spectrum, as seen from Figure 3, Omega–Pi should be the first choice. Nonetheless, this combination transmits light from the blue and red spectrum in the same way as well.

**Figure 4:**
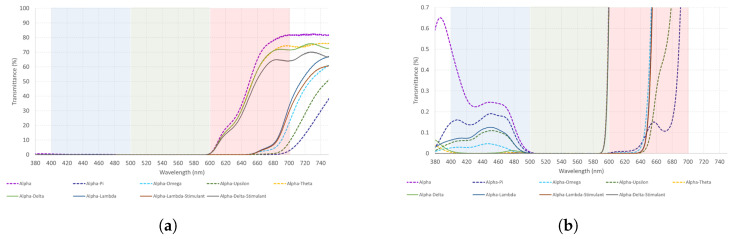
(**a**,**b**) The measured spectral transmittance (from 380 to 750 nm) of Alpha combinations in two different scales for its better understanding. Considering the vast number of Alpha combinations, the colors on the graphs are chosen arbitrarily and do not represent the colors perceived through each combination.

**Figure 5:**
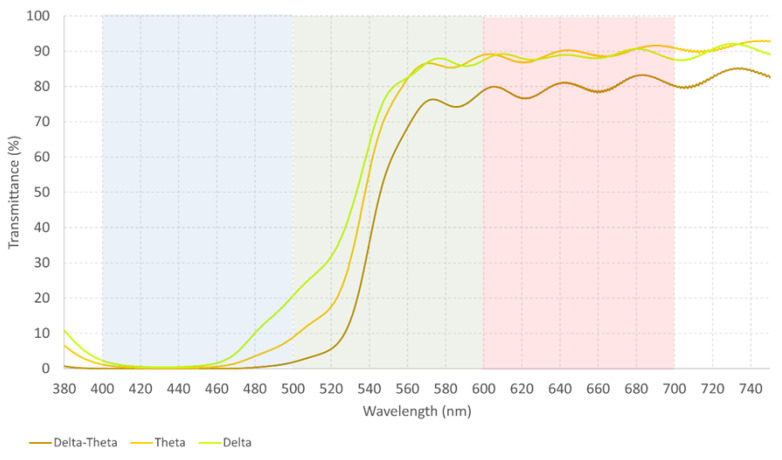
The measured spectral transmittance (from 380 to 750 nm) of the Delta–Theta filter. The colors on the graphs are chosen to approximately represent the colors perceived through each filter and its combination.

Alpha is a long-wavelength filter. Table 5 shows that Alpha–Delta and Alpha–Theta transmit light from the red spectrum almost the same way, followed by Alpha–Delta–S. However, when Delta and Theta are combined, transmittance increases. Even though there is a decrease in Alpha transmittance when combined with short-wavelength filters, it remains higher in the red than blue spectrum. See Figure 4a,b and Figure 5 for a more visual representation of these results.

Figure 4 illustrates that no significant changes are found between Alpha–Delta and Alpha–Theta combinations. They transmit light from the red spectrum almost exactly the same way and could be interchangeable. Nonetheless, if we are looking for a stronger reaction, Delta–Theta should be used as Figure 5 shows.

**Figure 6:**
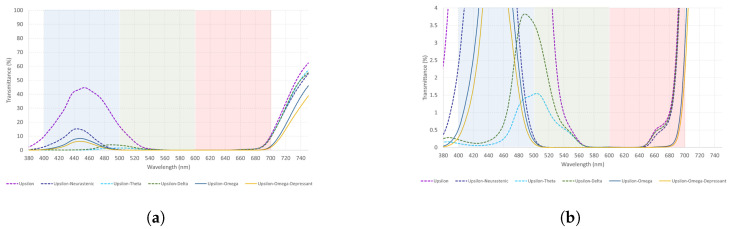
(**a**,**b**) The measured spectral transmittance (from 380 to 750 nm) of Upsilon combinations in two different scales for its better understanding. Considering the number of combinations, the colors on the graphs are chosen arbitrarily and do not represent the colors perceived through each combination.

Upsilon is a short-wavelength filter. From Table 6, it can be seen that even when combined with other short-wavelength filters, there is a higher light transmittance to the red than blue spectrum, Upsilon–Neurasthenic the best combination to transmit light from the blue spectrum, followed by Upsilon–Omega and Upsilon–O–D. A graphical representation of these results is illustrated in Figure 6a,b.

From Figure 6, it can be seen that Upsilon combinations have a higher light transmittance to the red than blue spectrum. Therefore, their use should not be recommended when the goal is to transmit light from the blue spectrum.

**Figure 7:**
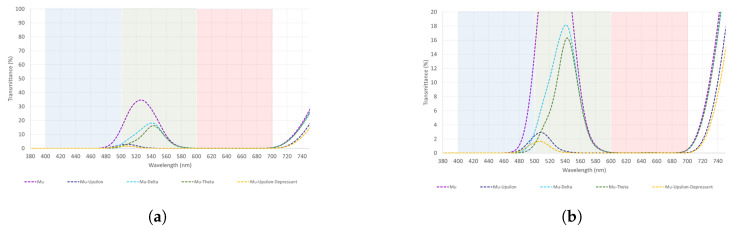
(**a**,**b**) The measured spectral transmittance (from 380 to 750 nm) of Mu combinations in two different scales for its better understanding. The colors on the graphs are chosen arbitrarily and do not represent the colors perceived through each combination.

Mu is a mid-wavelength filter. Results from Table 7 suggest that Mu–Delta is the best filter to transmit light to the green spectrum, followed by Mu–Theta. Additionally, these combinations transmit light from the red spectrum in almost the same way. Refer to Figure 7a,b, for a better visual understanding of the data.

As shown in Figure 7, Mu–Delta and Mu–Theta transmit light to the green and red spectrum in a very similar way. These combinations could be used in both cases interchangeably. Nevertheless, Mu–Delta should be preferred as transmittance from the green spectrum is higher than the red one.

### 2.2. Effect on the Three Cone Cells of the Retina and Their Relative Sensitivity to Each Wavelength

**Figure 8:**
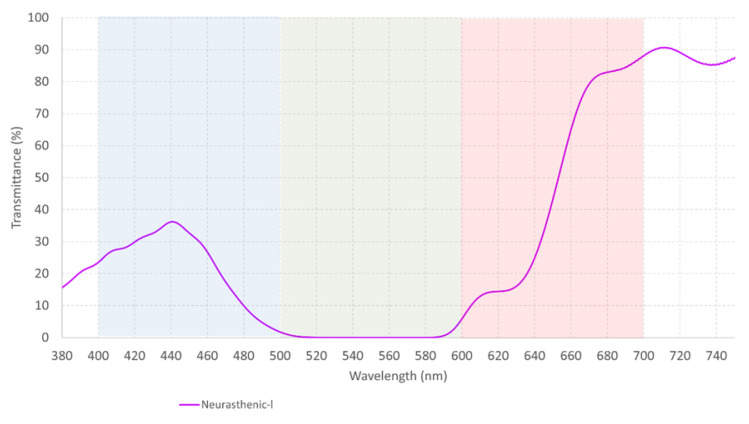
The measured spectral transmittance (from 380 to 750 nm) of the neurasthenic filter. The color on the graph represents the color perceived through the filter.

The examination consisted of analyzing the response of the retinal cone cells to all filters measured in this paper. The sensitivity functions of the cones used in the graphs (z, y and *x*-bar) were obtained from the International Commission on Illumination (ICN). These functions are described as CIE 1931 Color Matching Functions. This set of color-matching functions is representative of the color-matching properties of observers with normal color vision for visual field sizes of angular subtense from about $1^{0}$ to about $4^{0}$ for vision at photopic levels of adaptation. The eye sensitivity under their effect is presented in Table 8, Table 9, Table 10, Table 11 and Table 12. These values correspond to the percentage perceived by the cone at each wavelength interval under the effect of the filter. The five most representative wavelength ranges were chosen for the analysis to simplify the sensitivity functions of the three cone cells and only focus on the most relevant data. Likewise, Figure 8 was added to help visualize the maximum and minimum effect of the measured filters on each cone.

Table 8 illustrates that short-wavelength filters show a higher sensitivity to the blue cone cells (numbers in bold), followed by the red cones, except for the Pi filter, which has its second peak on the green cones. Long-wavelength filters have a more significant impact on the green cones, followed by the red cone cells, but for the Alpha filter, its higher peak on the red cones is followed by the green cones.

We can see in Table 9 how combined short-wavelength filters show a weak effect on the cone cells of the retina. There is a remarkable decrease in their power, Omega–Neurasthenic and Omega–Pi the only two combinations with a weak effect on the blue cone. Nevertheless, the percentage of the relative sensitivity on the green and red cones is insignificant.

Results from Table 10 indicate that when a long-wavelength filter (Alpha) is combined with a short-wavelength one (Omega, Upsilon, Pi, Lambda), the relative sensitivity on the three cone cells of the retina is insignificant. Only the Alpha–Delta and Alpha–Theta combinations show a weak effect on the red cone cell and even weaker on the green cone. When three long-wavelength filters are combined, the effect on the red cone decreases even more, as represented by the Alpha–Delta–S filter. Delta–Theta is the best combination, with a higher impact on the green and posteriorly the red cone cell.

Table 11 suggests that the Upsilon–Neurasthenic and Upsilon–Omega are the strongest combinations despite their weak impact on the blue cone cells. The more filters are combined, the weaker their effect on the retinal cone cells, as Upsilon–O–D represents.

Table 12 indicates that Mu–Delta and Mu–Theta are the only two combinations worth analyzing, despite their weak effect on the green cone cells and even weaker effect on the red ones. When a mid-wavelength filter is combined with a short-wavelength one, the effect on the cone cells of the retina is insignificant.

**Figure 9:**
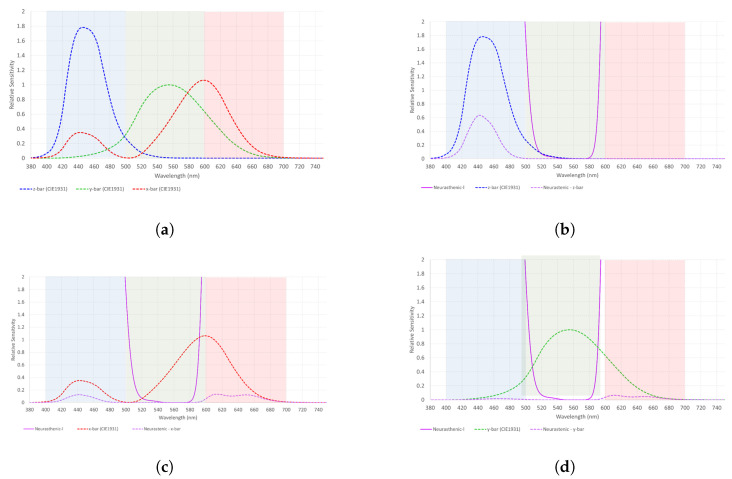
(**a**) The relative sensitivity of the blue, green, and red cone cells obtained from the International Commission on Illumination; (**b**) The relative sensitivity of the blue cone cells obtained from the International Commission on Illumination and how it changes under the effect of the neurasthenic filter. There is a diminished response of the blue cone cell with the filter on; (**c**) The relative sensitivity of the red cone cells obtained from the International Commission on Illumination and its diminished response with the neurasthenic filter on; and (**d**) The relative sensitivity of the green cone cells obtained from the International Commission on Illumination and how it reacts to the neurasthenic filter. There is a significant decrease on its value with the filter on.

As already mentioned, to simplify the understanding of the eye’s reaction (cone cells response) to each filter and their combinations, a graphical example is presented in Figure 8 and Figure 9a–d. The neurasthenic graphs were arbitrarily chosen for this purpose.

### 2.3. Comparison of Five Glass and Polymer Filters

**Figure 10:**
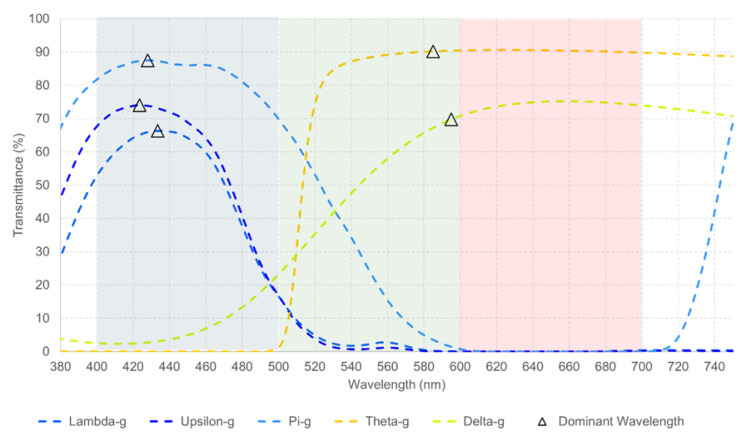
The measured spectral transmittance (from 380 to 750 nm) for the dominant wavelength of five monochromatic glass filters, with each dominant wavelength marked by a triangle. The colors shown in the graphs closely resemble the colors perceived through each filter. The dominant wavelength, defined as “the wavelength with the highest transmittance in the measured spectra”, falls within the ranges specified in the Visible Light Spectrum (Table 1) and corresponds to the perceived color of each filter.

**Figure 11:**
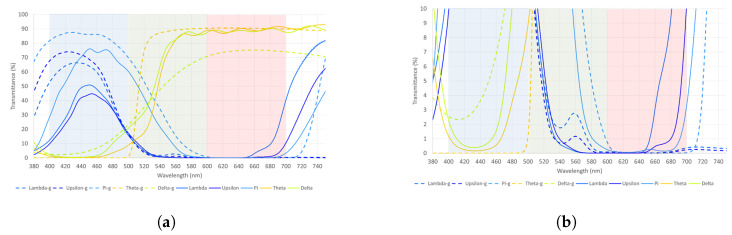
(**a**,**b**) The measured spectral transmittance (from 380 to 750 nm) of the five glass filters (dotted line) and their polymer counterparts (whole line) in two different scales. The colors on the graphs are chosen to approximately represent the colors perceived through each filter.

In clinical practice, two different devices are used to treat patients with visual dysfunction. The first uses glass filters (commonly used at the office for the evaluation protocol), and the other one (frequently used by patients at home during 20 consecutive days, the complete cycle of phototherapy) uses polymer filters. We considered it interesting to compare their wavelength, transmittance, and relative sensitivity to see their similarities and/or differences. These five short- and long-wavelength glass filters were arbitrarily chosen to be compared to the polymer ones. The characterization and properties of the five monochromatic glass filters chosen for this purpose are illustrated in Table 12, Table 13, Table 14 and Table 15 and Figure 10 and Figure 11.

Table 14 indicates that the Pi filter transmits light from the blue spectrum at a higher % than Upsilon and Lambda. Theta, on the other hand, transmits light from the red spectrum better than Delta. Moreover, some differences can be appreciated if these results are compared to those in Table 3. The most significant difference is the transmittance on the red spectrum when glass is compared to polymer filters. For Lambda and Upsilon, the transmittance of light to the red spectrum is insignificant. Additionally, glass filters show a greater transmittance in their corresponding wavelengths than polymer ones.

Data in Table 15 illustrate that short-wavelength filters have a significant impact on the blue cone cells and a second peak on the red cone, except for the Pi filter, with its second peak on the green cone cell. Likewise, the long-wavelength filters have a bigger effect on the green cone cells, followed by the red ones. If we compare these data with those of Table 8, the effect on the cone cells is higher for Theta, Lambda, and Pi but lower for Delta and Upsilon. Their transmittances and wavelengths are presented in Figure 11a,b to compare glass and polymer filters visually.

Figure 11 shows a higher transmittance rate for the glass filters compared to the polymers. Differences are greater for filters that transmit light from the blue spectrum when compared to the red spectrum. The significance of this in clinical practice remains unknown. It should be analyzed to measure whether there is an important change in the cortical networks of patients with visual dysfunctions when using glass or polymer filters.

Considering that light entering the visual system is transmitted to the retina and captured by its photoreceptors, Table 16 was added, recommending the principal filters and combinations to be considered when treating patients with visual dysfunctions.

Table 16 suggests that combinations could be reduced by half or more (considering that at least four combinations can be interchangeable), as the effect on the relative sensitivity of cone cells of the retina for the other half is insignificant. This would reduce the cost of the treatment and time and boost efficiency. Based on these new data and results from our previous studies using VEPs and qEEGs, a new protocol is being elaborated to increase light stimulation and phototherapy achievements.

## 3. Conclusions

This paper focused on measuring and characterizing eleven monochromatic filters and twenty-two combinations used empirically in the field of neuro-optometry as an essential part of the visual treatment of patients with visual dysfunctions. This is the first time that their properties were made available to visual health professionals, a crucial step towards the complete understanding of their physical characteristics to propose innovative and enhanced protocols to reduce costs and time and boost the effect of the treatment. One of the most relevant findings was that when two or three filters are combined, regardless of the wavelength, their transmittance and relative sensitivity on the retinal cone cells decrease. Therefore, combined filters may lose efficiency when used in treatments, suggesting that monochromatic filters should be preferred when looking for faster and bigger changes. Nonetheless, considering that filters are also used in patients with mild head trauma and/or patients with different neurological conditions, some combinations should be considered, because of their more attenuated effect on the cone cell response. This would help the visual system to react and recover at its own pace avoiding any disruption to a fragile brain. Additionally, most filters and combinations, regardless of the wavelength, transmit a considerable percentage of light from the red spectrum. These results suggest that the phototherapy treatment protocol used nowadays must be deeply revised, and the number of filters and combinations should be reduced to cut costs and time and boost efficiency.

## Figures and Tables

**Table 1:** Shows the range of the wavelength expressed in (nm) of the Visible Light Spectrum.

Visible Light Spectrum Color	Range of the Wavelength (nm)
Violet	380 to 440
Blue	440 to 480
Cyan	480 to 500
Green	500 to 560
Yellow	560 to 590
Orange	590 to 620
Red	620 to 780

**Table 2:** Represents the wavelength and transmittance of each monochromatic filter measured in this study.

Name of the Filter	Wavelength (nm)	Transmittance (%)
Neurasthenic	440.5	36.02
Omega	446.5	19.00
Depressant	445	79.57
Mu	526.5	34.76
Stimulant	592	89.17
Alpha	731	81.80
Delta	576	87.99
Theta	571	86.57
Upsilon	453.5	44.70
Lambda	450	50.87
Pi	461	76.05

**Table 3:** The average transmittance of each monochromatic filter in the seven basic wavelength ranges of the VLS.

Perceived Color	Wavelength (nm)	Neurasthenic (N)	Omega (Ω)	Depressant (D)	Mu (μ)	Stimulant (s)	Alpha (α)	Lambda (λ)	Pi (π)	Upsilon (υ)	Theta (θ)	Delta (δ)
Violet	360 to 440	22.55	6.71	60.21	0.01	1.44	0.39	17.52	30.15	13.89	3.68	6.12
Blue	440 to 480	25.37	15.01	78.13	0.14	0.58	0.23	45.97	74.24	41.54	0.99	2.81
Cyan	480 to 500	5.29	3.74	68.97	5.77	21.55	0.05	24.75	65.76	25.48	5.81	15.15
Green	500 to 560	0.21	0.16	43.29	25.73	72.25	<0.01	4.08	32.67	4.91	38.97	49.03
Yellow	560 to 590	0.06	<0.01	17.30	2.95	86.07	−	0.03	2.53	0.04	85.54	86.22
Orange	590 to 620	8.73	0.03	15.39	0.09	87.74	5.84	0.01	0.21	0.01	88.08	87.86
Red	620 to 780	71.97	34.89	63.63	11.30	89.11	69.47	40.25	18.19	25.95	90.50	89.29

The negative sign (−) for different measurements represents values below zero, with insignificant impact on the visual system.

**Table 4:** The average transmittance of Omega combinations in seven different wavelength ranges of the VLS.

Perceived Color	Wavelength (nm)	Omega–Neurasthenic	Omega–Theta	Omega–Delta	Omega–Pi
Violet	360 to 440	1.92	0.06	0.11	3.30
Blue	440 to 480	4.02	0.11	0.31	10.96
Cyan	480 to 500	0.22	0.18	0.49	2.42
Green	500 to 560	<0.01	0.02	0.04	0.08
Yellow	560 to 590	−	<0.01	<0.01	−
Orange	590 to 620	<0.01	0.02	0.02	−
Red	620 to 780	30.21	31.40	31.16	13.24

The negative sign (−) for different measurements represents values below zero, with insignificant impact on the visual system.

**Table 5:** The average transmittance of Alpha combinations in seven basic wavelength ranges of the VLS. The Delta–Theta filter is added to this table as it transmits light from the red spectrum.

Perceived Color	Wavelength (nm)	Alpha–Omega	Alpha–Upsilon	Alpha–Pi	Alpha–Theta	Alpha–Delta	Alpha–Lambda	Alpha–Lambda–S	Alpha–Delta–S	Delta–Theta
Violet	360 to 440	0.02	0.04	0.10	0.01	0.03	0.06	−	−	0.53
Blue	440 to 480	0.03	0.09	0.17	<0.01	< 0.01	0.10	−	−	0.05
Cyan	480 to 500	<0.01	0.02	0.04	<0.01	<0.01	0.02	<0.01	<0.01	0.93
Green	500 to 560	−	−	<0.01	−	−	−	−	−	24.72
Yellow	560 to 590	−	−	−	−	−	−	−	−	74.26
Orange	590 to 620	<0.01	−	<0.01	5.15	5.16	<0.01	−	4.63	78.06
Red	620 to 780	28.56	21.39	15.10	63.11	62.38	33.15	29.93	56.61	81.38

The negative sign (−) for different measurements represents values below zero, with insignificant impact on the visual system.

**Table 6:** Illustrates the average transmittance of Upsilon combinations in the seven wavelength ranges of the VLS.

Perceived Color	Wavelength (nm)	Upsilon–Neurasthenic Transmittance (%)	Upsilon–Theta	Upsilon–Omega	Upsilon–Delta	Upsilon–O–D
Violet	360 to 440	4.13	0.10	1.63	0.19	1.13
Blue	440 to 480	10.71	0.39	6.28	1.09	4.68
Cyan	480 to 500	1.45	1.40	1.00	3.71	0.67
Green	500 to 560	0.03	0.84	0.02	1.46	0.01
Yellow	560 to 590	−	0.03	−	0.03	−
Orange	590 to 620	<0.01	0.01	−	0.01	−
Red	620 to 780	22.79	23.58	17.97	23.33	15.13

O, Omega; D, Depressant. The negative sign (−) for different measurements represents values below zero, with insignificant impact on the visual system.

**Table 7:** The average transmittance of Mu combinations in seven different wavelength ranges of the VLS.

Perceived Color	Wavelength (nm)	Mu–Upsilon	Mu–Upsilon–D	Mu–Delta	Mu–Theta
Violet	360 to 440	<0.01	−	−	−
Blue	440 to 480	0.05	0.04	0.01	<0.01
Cyan	480 to 500	1.30	0.84	0.99	0.39
Green	500 to 560	1.24	0.64	11.94	9.09
Yellow	560 to 590	<0.01	−	2.53	2.53
Orange	590 to 620	−	−	0.08	0.08
Red	620 to 780	7.44	6.29	10.15	10.31

D, Depressant. The negative sign (−) for different measurements represents values below zero, with insignificant impact on the visual system.

**Table 8:** The response of the three cone cells of the retina to each monochromatic filter. The *z*, *y*, and *x*-bars represent “the sensitivity functions of the blue, green and red cone cells” as determined by the ICN. The percentage of “relative sensitivity” was calculated for the five most representative wavelength ranges of the VLS.

		Neurasthenic	Omega	Depressant	Mu	Stimulant	Alpha	Lambda	Pi	Upsilon	Theta	Delta
		Percentage of Relative Sensitivity (%)										
Blue Cone	*z*-bar											
360 to 400 nm	0.49	0.10	0.02	0.27	−	<0.01	<0.01	0.04	0.09	0.03	0.01	0.03
400 to 500 nm	95.24	**25.31**	**13.48**	**72.54**	0.49	1.90	0.22	**40.10**	**66.10**	**35.68**	1.02	2.75
500 to 600 nm	4.26	0.02	0.02	2.29	1.05	2.53	<0.01	0.37	2.02	0.43	0.81	1.33
600 to 700 nm	0.01	0.01	−	<0.01	−	0.01	<0.01	−	−	−	0.01	0.01
700 to 780 nm	0	0	0	0	0	0	0	0	0	0	0	
Green Cone	*y*-bar											
360 to 400 nm	<0.01	<0.01	<0.01	<0.01	−	−	−	<0.01	<0.01	<0.01	−	<0.01
400 to 500 nm	6.64	0.79	0.49	4.75	0.26	0.96	0.01	2.10	4.54	2.04	0.28	0.72
500 to 600 nm	75.64	0.22	0.04	23.01	**12.08**	**60.83**	0.01	1.25	13.14	1.55	**47.12**	**51.26**
600 to 700 nm	17.67	3.42	0.06	3.22	0.01	15.51	3.46	0.08	0.02	0.01	15.63	15.63
700 to 780 nm	0.06	0.05	0.03	0.05	<0.01	0.05	0.05	0.03	<0.01	0.01	0.05	0.05
Red Cone	x-bar											
360 to 400 nm	0.10	0.02	<0.01	0.06	−	<0.01	<0.01	0.01	0.02	0.01	<0.01	0.01
400 to 500 nm	16.60	4.74	2.48	12.71	0.03	0.14	0.04	7.14	11.47	6.29	0.12	0.32
500 to 600 nm	44.37	0.26	<0.01	9.28	3.19	38.13	0.01	0.11	2.73	0.14	35.43	36.22
600 to 700 nm	38.76	8.16	0.16	7.35	0.01	34.06	**8.50**	0.22	0.05	0.03	34.30	34.28
700 to 780 nm	0.16	0.14	0.07	0.13	0.01	0.14	0.13	0.09	0.02	0.04	0.14	0.14

The negative sign (−) for different measurements represents values below zero, with insignificant impact on the visual system.

**Table 9:** The response of the retinal cone cells to every Omega combination. The *z*, *y*, and *x*-bars represent “the sensitivity functions of the blue, green and red cone cells” as determined by the ICN. The percentage of “relative sensitivity” was calculated for the five wavelength ranges of the VLS.

		Omega–Neurasthenic	Omega–Theta	Omega–Delta	Omega–Pi
		Percentage of Relative Sensitivity (%)			
Blue Cone	*z*-bar				
360 to 400 nm	0.49	<0.01	<0.01	<0.01	<0.01
400 to 500 nm	95.24	3.87	0.08	0.22	9.39
500 to 600 nm	4.26	<0.01	<0.01	<0.01	0.01
600 to 700 nm	0.01	−	−	−	−
700 to 780 nm	0	0	0	0	0
Green Cone	*y*-bar				
360 to 400 nm	<0.01	−	−	−	−
400 to 500 nm	6.64	0.09	0.01	0.02	0.34
500 to 600 nm	75.64	<0.01	<0.01	0.01	0.02
600 to 700 nm	17.67	0.04	0.05	0.05	<0.01
700 to 780 nm	0.06	0.02	0.02	0.02	<0.01
Red Cone	*x*-bar				
360 to 400 nm	0.10	<0.01	−	<0.01	<0.01
400 to 500 nm	16.60	0.74	0.01	0.03	1.72
500 to 600 nm	44.37	−	<0.01	<0.01	<0.01
700 to 780 nm	0.16	0.06	0.06	0.06	0.01

The negative sign (−) for different measurements represents values below zero, with insignificant impact on the visual system.

**Table 10:** The response of the three cone cells of the retina to all alpha combinations. The *z*, *y*, and *x*-bars represent “the sensitivity functions of the blue, green and red cone cells” as determined by the ICN. The percentage of “relative sensitivity” was calculated for the five wavelength ranges of the VLS.

		Alpha–Omega	Alpha–Upsilon	Alpha–Pi	Alpha–Theta	Alpha–Delta	Alpha–Lambda	Alpha–Lambda-S	Alpha–Delta-S	Delta–Theta
		Percentage of Relative Sensitivity (%)								
Blue Cone	*z*-bar									
360 to 400 nm	0.49	<0.01	<0.01	<0.01	−	<0.01	<0.01	−	−	<0.01
400 to 500 nm	95.24	0.03	0.08	0.15	<0.01	<0.01	0.09	−	−	0.10
500 to 600 nm	4.26	−	−	−	−	−	−	−	−	0.35
600 to 700 nm	0.01	−	−	−	<0.01	<0.01	−	−	<0.01	0.01
700 to 780 nm	0	0	0	0	0	0	0	0	0	0
Green Cone	*y*-bar									
360 to 400 nm	<0.01	−	−	−	−	−	−	−	−	−
400 to 500 nm	6.64	<0.01	<0.01	0.01	<0.01	<0.01	<0.01	−	−	0.04
500 to 600 nm	75.64	−	−	−	0.01	0.01	−	−	0.01	36.87
600 to 700 nm	17.67	0.04	0.01	0.01	3.14	3.13	0.06	0.05	2.81	13.94
700 to 780 nm	0.06	0.02	0.01	0.01	0.04	0.04	0.03	0.02	0.04	0.05
Red Cone	*x*-bar									
360 to 400 nm	0.10	−	−	<0.01	−	−	−	−	−	<0.01
400 to 500 nm	16.60	0.01	0.01	0.03	<0.01	<0.01	0.02	−	−	0.01
500 to 600 nm	44.37	−	−	−	0.01	0.01	−	−	0.01	29.87
600 to 700 nm	38.76	0.11	0.02	0.01	7.70	7.68	0.16	0.14	6.89	30.58
700 to 780 nm	0.16	0.06	0.03	0.02	0.12	0.12	0.07	0.07	0.11	0.13

S, Stimulant. The negative sign (−) for different measurements represents values below zero, with insignificant impact on the visual system.

**Table 11:** The response of the retinal cone cells to each Upsilon combination. The *z*, *y*, and *x*-bars represent “the sensitivity functions of the blue, green and red cone cells” as determined by the ICN. The percentage of “relative sensitivity” was calculated for the five wavelength ranges of the VLS.

		Upsilon–Neurasthenic	Upsilon–Theta	Upsilon–Omega	Upsilon–Delta	Upsilon–O–D
		Percentage of Relative Sensitivity (%)				
Blue Cone	*z*-bar					
360 to 400	0.49	0.01	<0.01	<0.01	<0.01	<0.01
400 to 500	95.24	9.72	0.33	5.28	0.90	3.90
500 to 600	4.26	<0.01	0.05	<0.01	0.11	<0.01
600 to 700	0.01	−	−	−	−	−
700 to 780	0	0	0	0	0	0
Green Cone	*y*-bar					
360 to 400 nm	<0.01	−	−	−	−	−
400 to 500 nm	6.64	0.29	0.07	0.18	0.18	0.13
500 to 600 nm	75.64	0.01	0.32	<0.01	0.52	<0.01
600 to 700 nm	17.67	0.01	0.01	<0.01	0.01	<0.01
700 to 780 nm	0.06	0.01	0.01	0.01	0.01	0.01
Red Cone	*x*-bar					
360 to 400 nm	0.10	<0.01	<0.01	<0.01	<0.01	<0.01
400 to 500 nm	16.60	1.81	0.04	0.97	0.12	0.72
500 to 600 nm	44.37	−	0.06	−	0.08	−
600 to 700 nm	38.76	0.02	0.03	<0.01	0.03	<0.01
700 to 780 nm	0.16	0.04	0.04	0.02	0.04	0.02

O, Omega; D, Depressant. The negative sign (−) for different measurements represents values below zero, with insignificant impact on the visual system.

**Table 12:** The response of the three cone cells of the retina to all Mu combinations. The *z*, *y*, and *x*-bars represent “the sensitivity functions of the blue, green and red cones” as determined by the ICN. The percentage of “relative sensitivity” was calculated for the five wavelength ranges of the VLS.

		Mu–Upsilon	Mu–Upsilon–D	Mu–Delta	Mu–Theta
		Percentage of Relative Sensitivity (%)			
Blue Cone	*z*-bar				
360 to 400 nm	0.49	−	−	−	−
400 to 500 nm	95.24	0.12	0.08	0.07	0.03
500 to 600 nm	4.26	0.09	0.05	0.33	0.20
600 to 700 nm	0.01	−	−	−	−
700 to 780 nm	0	0	0	0	0
Green Cone	*y*-bar				
360 to 400 nm	<0.01	−	−	−	−
400 to 500 nm	6.64	0.06	0.04	0.05	0.02
500 to 600 nm	75.64	0.41	0.20	6.40	5.22
600 to 700 nm	17.67	−	−	0.01	0.01
700 to 780 nm	0.06	<0.01	<0.01	<0.01	<0.01
Red Cone	*x*-bar				
360 to 400 nm	0.10	−	−	−	−
400 to 500 nm	16.60	0.01	0.01	<0.01	<0.01
500 to 600 nm	44.37	0.04	0.02	2.16	1.95
600 to 700 nm	38.76	−	−	0.01	0.01
700 to 780 nm	0.16	<0.01	<0.01	0.01	0.01

D, Depressant. The negative sign (−) for different measurements represents values below zero, with insignificant impact on the visual system.

**Table 13:** The wavelength and transmittance of five monochromatic glass filters measured in this study.

Name of the Filter	Wavelength (nm)	Transmittance (%)
Delta	595	69.75
Theta	585	90.17
Upsilon	423.5	74.05
Lambda	433.5	66.28
Pi	428	87.51

**Table 14:** The average transmittance of five monochromatic glass filters in the seven basic wavelength ranges of the VLS.

Perceived Color	Wavelength (nm)	Lambda	Upsilon	Pi	Theta	Delta
		Transmittance (%)				
Violet	360 to 440	45.53	58.56	75.15	<0.01	3.34
Blue	440 to 480	57.01	61.42	85.26	<0.01	7.36
Cyan	480 to 500	26.13	27.60	76.08	0.13	17.90
Green	500 to 560	5.02	3.99	43.53	68.15	41.12
Yellow	560 to 590	1.20	0.51	7.59	89.79	63.72
Orange	590 to 620	0.08	0.021	0.65	90.48	71.45
Red	620 to 780	0.22	0.12	22.12	89.59	73.03

**Table 15:** The response of the retinal cone cells to these five monochromatic glass filters. The *z*, *y*, and *x*-bars represent “the sensitivity functions of the blue, green and red cones” as determined by the ICN. The percentage of “relative sensitivity” was calculated for the five wavelength ranges of the VLS.

		Lambda	Upsilon	Pi	Theta	Delta
		Percentage of Relative Sensitivity (%)				
Blue Cone	*z*-bar					
360 to 400 nm	0.49	0.22	0.29	0.37	−	0.01
400 to 500 nm	95.24	54.92	59.97	81.09	0.01	6.29
500 to 600 nm	4.26	0.39	0.36	2.47	1.68	1.36
600 to 700 nm	0.01	−	−	−	0.01	0.01
700 to 780 nm	0	0	0	0	0	0
Green Cone	*y*-bar					
360 to 400 nm	<0.01	<0.01	<0.01	<0.01	−	−
400 to 500 nm	6.64	2.44	2.61	5.26	0.01	0.95
500 to 600 nm	75.64	2.05	1.37	19.33	61.77	39.80
600 to 700 nm	17.67	0.01	<0.01	0.03	15.99	12.94
700 to 780 nm	0.06	<0.01	<0.01	<0.01	0.05	0.04
Red Cone	*x*-bar					
360 to 400 nm	0.10	0.04	0.06	0.08	−	<0.01
400 to 500 nm	16.60	10.00	10.95	14.24	<0.01	0.93
500 to 600 nm	44.37	0.54	0.24	5.16	39.52	27.38
600 to 700 nm	38.76	0.02	0.01	0.05	35.09	28.47
700 to 780 nm	0.16	<0.01	<0.01	0.01	0.14	0.12

The negative sign (−) for different measurements represents values below zero, with insignificant impact on the visual system.

**Table 16:** The effect of monochromatic and combined filters on the relative sensitivity of cone cells of the retina, identified by the number of “x”.

Impact on the Cone Cells of the Retina			
Filters	Blue cone	Green cone	Red cone
Depressant	xxxxxx	xxx	xxx
Pi	xxxxx	xx	xx
Lambda	xxxx		x
Upsilon	xxx		x
Neurasthenic	xx		x
Omega	x		
Mu		x	
Stimulant	xxxxxx		xxxxxx
Delta	xxxxx		xxxxx
Theta	xxxx		xxxx
Alpha			x
Combined filter			
Upsilon–Neurasthenic	xxxxx		
Omega–Pi	xxxx		
Upsilon–Omega	xxx		
Upsilon–O–D	xx		
Omega–Neurasthenic	x		
Delta–Theta		xxxxx	
Mu–Delta		xxxx	
Mu–Theta		xxx	
Alpha–Theta		xx	
Alpha–Delta		xx	
Delta–Theta			xxx
Alpha–Theta			xx
Alpha–Delta			xx
Alpha–Delta–S			x

## Data Availability

The database is available upon request to the authors.
